# The two faces of synaptic failure in *App*^*NL-G-F*^ knock-in mice

**DOI:** 10.1186/s13195-020-00667-6

**Published:** 2020-08-24

**Authors:** Amira Latif-Hernandez, Victor Sabanov, Tariq Ahmed, Katleen Craessaerts, Takashi Saito, Takaomi Saido, Detlef Balschun

**Affiliations:** 1 Brain and Cognition, KU Leuven, Tiensestraat 102, Box 3714, 3000 Leuven, Belgium; 2 Present Address: Neurology and Neurological Sciences, Stanford Medicine, Stanford, USA; 3 Leuven Brain Institute, KU Leuven, Leuven, Belgium; 4 Present Address: Qatar Biomedical Research Institute, Ar-Rayyan, Qatar; 5 Laboratory for the Research of Neurodegenerative Diseases, VIB Center for the Biology of Disease, Leuven, Belgium; 6 Laboratory for Proteolytic Neuroscience, RIKEN Center for Brain Science, Wako-shi, Saitama Japan; 7 Present Address: Department of Neurocognitive Science, Nagoya City University Graduate School of Medical Science, Nagoya, Aichi Japan

**Keywords:** App knock-in mice, Long-term potentiation, Long-term depression, Miniature synaptic currents, Presynaptic glutamatergic and GABAergic upregulation, Electrophysiological phenotyping

## Abstract

**Background:**

Intensive basic and preclinical research into Alzheimer’s disease (AD) has yielded important new findings, but they could not yet been translated into effective therapies. One of the reasons is the lack of animal models that sufficiently reproduce the complexity of human AD and the response of human brain circuits to novel treatment approaches. As a step in overcoming these limitations, new App knock-in models have been developed that avoid transgenic APP overexpression and its associated side effects. These mice are proposed to serve as valuable models to examine Aß-related pathology in “preclinical AD.”

**Methods:**

Since AD as the most common form of dementia progresses into synaptic failure as a major cause of cognitive deficits, the detailed characterization of synaptic dysfunction in these new models is essential. Here, we addressed this by extracellular and whole-cell patch-clamp recordings in *App*^*NL-G-F*^ mice compared to *App*^*NL*^ animals which served as controls.

**Results:**

We found a beginning synaptic impairment (LTP deficit) at 3–4 months in the prefrontal cortex of *App*^*NL-G-F*^ mice that is further aggravated and extended to the hippocampus at 6–8 months. Measurements of miniature EPSCs and IPSCs point to a marked increase in excitatory and inhibitory presynaptic activity, the latter accompanied by a moderate increase in postsynaptic inhibitory function.

**Conclusions:**

Our data reveal a marked impairment of primarily postsynaptic processes at the level of synaptic plasticity but the dominance of a presumably compensatory presynaptic upregulation at the level of elementary miniature synaptic function.

## Introduction

There is a general consensus on two major histopathological characteristics of AD, extracellular amyloid plaques, consisting of fibrils and non-fibrillar forms of the polypeptide amyloid ß (Aß) and neurofibrillary tangles (NFTs), and intracellular aggregates that are composed of hyperphosphorylated forms of the tau protein [1]. Aß originates from the sequential endoproteolytic cleavage of amyloid precursor protein (APP) resulting in several forms of Aß of which the 42-residue Aß_42_ has the strongest propensity to form aggregates and the highest cellular toxicity [2–4].

The discovery of genetically inherited, early-onset, familial forms of AD (FAD) in the 1990s of the last century made Aß the primary focus of AD research for more than two decades. FAD patients carry missense mutations in the genes encoding amyloid precursor protein (*APP*), presenilin 1 (*PSEN1*) and presenilin 2 (*PSEN2*), respectively (see [5] for references). These mutations result invariably in the generation of longer or more aggregating forms of Aβ. As this seems sufficient for the full development of similar clinical features as the much more common spontaneous, late-onset AD (LOAD) [6–8], it is important to investigate the effect of Aß on the cellular manifestation of the disease [9].

Despite the fact that FAD contributes to less than 0.1% of AD cases, its discovery has boosted the generation of a variety of transgenic mouse models that carry a combination of these human mutations and replicate key aspects of human AD, like amyloid deposition and progressing cognitive decline [10, 11]. These “first-generation” transgenic AD mouse models have been invaluable for delineating multifarious molecular mechanisms of disease onset and progression, but they share the limitation that the proteolytic processing of overexpressed APP results not only in the overproduction of Aß, but also of some other APP fragments. Thus, in these mice, pathological changes per se cannot be clearly attributed to an increased Aβ production since they could also be due to the (patho) physiological effects of one or several of the other APP fragments [12]. To overcome these drawbacks of APP overexpression, a new generation of mouse models of sporadic AD has been developed by the Saido laboratory [13] using a knock-in strategy to introduce the Swedish mutation, which increases all Aß species, into the APP gene, together with either the Beyreuther/Iberian mutation or the Beyreuther/Iberian plus the Arctic mutation [13, 14]. These new models, denominated as *App*^*NL-F*^ and *App*^*NL-G-F*^, express normal APP levels but develop robust Aß pathology resulting in synaptic degeneration and memory impairments [14]. More specifically, *App*^*NL-F*^ *mice* develop high levels of Aß_42_ and a high Aß42/40 ratio without changes in the expression of APP and other fragments (except a shifted ratio of CTF-α/CTF-ß). Addition of the Arctic mutation to *App*^*NL-F*^ resulted in mice (*App*^*NL-G-F*^) that progress threefold faster to a more severe AD pathology and cognitive deficits compared to *App*^*NL-F*^ mice [12, 13]. Comparative studies of these mice with first-generation transgenic APP models confirmed the hypothesis that some findings with the latter are likely to be due to “side effects” of overexpressing APP and non-Aß fragments rather than the increased levels of Aß or Aß42/40 [12, 13, 15]. With regard to the ongoing characterization of cognitive performance of *App*^*NL-F*^ and *App*^*NL-G-F*^ mice, memory deficits were reported at 6 months in *App*^*NL-G-F*^ and 18 months in *App*^*NL-F*^ mice [13, 16–18]. However, some of the memory deficits observed at 6 months of age in *App*^*NL-G-F*^ mice have been subtle [17] or could not be reproduced by others [19]. Together, these data led to the conclusion that “App knock-in mice should be considered models of preclinical AD” [12].

Research on preclinical models of AD and their characterization requires sensitive tools to detect subtle indications of incipient pathology. Given that AD as the most common form of dementia [1, 20] is the integrative result of a complex interplay of multiple multicellular pathophysiological processes [9] with synaptic failure as a major downstream pathological deterioration [21, 22], early signs of pathological changes are likely to be discernible at the level of synapses. Electrophysiological measures of synaptic transmission and plasticity are sensitive to even minor changes in pre- and postsynaptic functions [23–27] and therefore meet these requirements optimally.

However, an evaluation of *App*^*NL-G-F*^ mice at the synaptic level, i.e., the primary locus of the pathological deterioration of cognition, is still lacking. To address this, we used in the current study long-term extracellular recordings in acute slices of the prefrontal cortex (PFC) and the hippocampus (HC) to evaluate activity-dependent synaptic changes at two different stages of AD pathology, 3–4 and 6–8 months. Whole-cell patch-clamp recordings of mEPSCs and mIPSCs at the age of 6–8 months, i.e., when an almost saturated amyloidosis is present in these mice [13], complemented these experiments. Measurements of soluble and insoluble Aß_40_ and Aß_42_ tissue levels served as an indicator of the progression of Aß pathology.

We found first signs of synaptic impairment already at 3–4 months of age in *App*^*NL-G-F*^ mice, becoming overt as faster decay of LTP in PFC. With further progression of pathology at 6–8 months, PFC LTP was severely impaired, paralleled by a marked reduction in basal synaptic transmission. In contrast, in the hippocampal CA1 region, basal synaptic transmission, short-term plasticity, LTP, and LTD were inconspicuous at 3–4 months, but at 6–8 months, LTP was clearly impaired and short-term plasticity (paired-pulse ratio at 10 ms interpulse interval) reduced. No changes were found in basal synaptic transmission and LTD. Whole-cell patch-clamp recordings at 6–8 months, the age of pronounced synaptic pathology, revealed increased mEPSC and mIPSC frequency pointing to an enhanced presynaptic activity. The increase in mIPSC amplitude suggested that the increase in GABAergic transmission included also postsynaptic mechanisms.

To our knowledge, this is the first electrophysiological characterization of hippocampal and prefrontal synaptic functioning of these second-generation AD models, which are expected to become a standard for identifying mechanisms and pathways upstream and downstream of Aβ amyloidosis [13].

## Materials and methods

### Animals

The housing conditions and procedures to prepare acute brain slices were approved by the KU Leuven Ethical Committee and in accordance with European Directive 2010/63/EU. Homozygous female *App*^*NL*^ and *App*^*NL-G-F*^ mice were derived from the breeding colony established in the laboratory of Bart De Strooper. The *App*^*NL-G-F*^ mice co-express the Swedish (KM670/671NL), the Beyreuther/Iberian (I716F), and the Arctic (E693G) mutations. *App*^*NL*^ mice that only express the Swedish mutation and do not develop any significant pathology [13, 14] served as controls. Saito et al. and Mehla et al. reported an age-dependent Aβ amyloidosis in homozygous *App*^*NL-G-F*^ mice [13, 18]. Notably, in the study of Saito et al., the cortical deposition began by 2 months and was almost saturated by 7 months, while Mehla et al. detected significant deposition in the cortex and hippocampus at 6 months, which peaked at 9–12 months of age. Sacher et al. came to similar results as Mehla et al. using a longitudinal PET imaging of amyloid load with 18F-florbetaben as tracer [28].

### Electrophysiological recordings

#### Extracellular long-term recordings in the hippocampal CA1 region

Electrophysiological recordings were performed in hippocampal slices as previously described [29]. Briefly, 3–4 and 6–8 months old female *App*^*NL-G-F*^ and *App*^*NL*^ were tested. Animals were killed by cervical dislocation, and the hippocampus was rapidly dissected out into ice-cold (4 °C) artificial cerebrospinal fluid (ACSF), saturated with carbogen (95% O_2_/5% CO_2_). ACSF consisted of (in mM) the following: 124 NaCl, 4.9 KCl, 24.6 NaHCO_3_, 1.20, KH_2_PO_4_, 2.0 CaCl_2_, 2.0 MgSO_4_, 10.0 glucose, pH 7.4. Transverse slices (400 μm thick) were prepared from the dorsal area of the right hippocampus with a tissue chopper and placed into a submerged-type chamber, where they were maintained at 32 °C and continuously perfused with carbogen-saturated ACSF at a flow rate of 2.5 ml/min. After 90 min of incubation, one slice was arbitrarily selected and a tungsten electrode was placed in CA1 stratum radiatum. For recording of field excitatory postsynaptic potentials (fEPSPs), a glass electrode (filled with ACSF, 3–7MΩ) was placed in the stratum radiatum, opposite the stimulating electrode. The time course of the field EPSP was measured as the descending slope function for all sets of experiments. After input/output (I/O) curves had been established, the stimulation strength was adjusted to elicit a fEPSP slope of 35% of the maximum and kept constant throughout the experiment. For paired-pulse ratios, responses to two impulses given at an interval of 10, 20, 50,100, 200, or 500 ms were recorded as described in [30, 31]. During baseline recording, 3 single stimuli (0.1 ms pulse width; 10 s interval) were measured every 5 min and averaged for the 60-min fEPSP values. To induce L-LTP, three theta burst stimuli (TBS, separated by 10 min, 0.2 ms pulse width) were applied. L-LTD was generated by three trains of low-frequency stimulation (LFS) at 2 Hz for 10 min (0.2 ms pulse width) [29, 30, 32].

#### Extracellular long-term recordings in medial PFC

Electrophysiological recordings were performed in coronal PFC slices, cut at 1.5–2.5 mm rostral from bregma as described in [33]. Animals were killed by cervical dislocation, and the whole brain was rapidly dissected into ice-cold preoxygenated artificial cerebrospinal fluid (ACSF) consisting of (in mM) 124 NaCl, 4.9 KCl, 2.5 CaCl_2_, 1.3 MgSO_4_, 1.2 NaH_2_PO_4_, 25.6 NaHCO_3_, and 16.6 glucose, gassed with 95% O_2_/5% CO_2_, at pH 7.4. Usually, two slices (400 μm thick) were prepared per mouse using a custom-made tissue chopper, and incubated for 1 h at room temperature before being placed in a submerged-type four-chamber recording system (Campden Instruments LTD, Loughborough, Leics., UK), and maintained there at 32 °C and a flow rate of 1.8 to 2 ml/min/chamber. In all experiments, custom-made monopolar tungsten electrodes were used for stimulation and ACSF-filled glass electrodes (5–7 MΩ resistance) for recording of field excitatory postsynaptic potentials (fEPSPs). The initial slope of the fEPSPs served as a measure of this potential. To assess basic properties of synaptic responses, I/O curves were established by stimulation with 30 to 90 μA constant currents. The stimulation strength was adjusted to evoke a fEPSP slope of 40% of the maximum and kept constant throughout the experiment. During baseline recording, three single stimuli (0.1 ms pulse width; 10 s interval) were measured every 5 min. Once a stable baseline was established, LTP was induced by 4 episodes of high-frequency stimulation (HFS) at 100 Hz for 1 s, with 5 min interval between consecutive episodes. The sample sizes mentioned for recordings always reflect the number of animals and not slices used.

#### Whole-cell patch-clamp recordings of CA1 pyramidal neurons

Postsynaptic currents from single CA1 pyramidal cells were recorded in transverse hippocampal slices (400 μm thick) as described elsewhere [34]. Slices were prepared using a vibratome (Microm HM 650 V, Thermo Scientific, Waltham, MA, USA) and were placed after the cutting for about 90 min in an incubation chamber containing ACSF (in mM: 124 NaCl, 4.9 KCl, 1.2 NaH_2_PO_4_, 25.6 NaHCO_3_, 2.0 CaCl_2_, 2.0 MgSO_4_, 10.0 glucose, pH 7.3–7.4) continuously perfused with 95%O_2_/5%CO_2_ at 32 °C.

Whole-cell voltage clamp recordings were performed at room temperature using a MultiClamp 700B patch-clamp amplifier, and data were collected using pClamp software (Axon Instruments, Union City, CA, USA). Recording electrodes pulled from borosilicate glass (World Precision Instruments) were filled with a solution containing the following (in mM): 135.0 CsMeSO_4_, 4.0 NaCl, 4.0 Mg-ATP, 0.5 EGTA-Na, 0.3 Na-GTP, 10.0 K-HEPES, 5.0 QX-314; pH 7.3 (pipette resistance 3–5 MΩ). Access resistance was 10–20 MΩ and was then compensated to 75%. Only neurons with the input resistance changing < 25% during the recordings were included in the study.

Based on reversal potential, miniature excitatory and inhibitory postsynaptic currents (mEPSCs and mIPSCs) were mostly measured consecutively from the same neurons (e.g., [35–37]). That is, mEPSCs were first recorded at the reversal potential for GABA_A_ receptor-mediated events (− 60 mV), and mIPSCs were recorded at the reversal potential for glutamatergic currents (+ 10 mV) with tetrodotoxin (1 μM) present in the bath medium. To verify that mEPSCs were indeed glutamatergic, they were blocked by 20 μM 6-cyano-7-nitroquinoxaline-2,3-dione (CNQX) and 10 μM d-aminophosphonovalerate (D-APV) at the end of the experiments. Similarly, mIPSCs could be blocked by 100 μM picrotoxin, a GABA_A_ receptor antagonist.

Data were low-pass filtered at 2 kHz and acquired at 10 kHz using Digidata 1440 and pClamp 10 software. Off-line analysis of mEPSCs and mIPSCs was performed using MiniAnalysis software (v.6.0.7, Synaptosoft, Decatur, GA, USA).

### Aβ_40_ and Aβ_42_ level quantification

Different brain regions (hippocampus, neocortex, and cerebellum) of *App*^*NL*^ and *App*^*NL-G-F*^ mice were dissected after transcardial perfusion with ice-cold phosphate-buffered saline (PBS). Tissue was homogenized in tissue protein extraction reagent (Pierce) supplemented with complete protease inhibitors (Roche). The homogenates were centrifuged at 4 °C for 1 h at 100,000×*g* (*Beckman TLA 100.4 rotor*), and the supernatants were used for ELISA. To assess the GuHCl-soluble Aβ fraction of the tissue, we used a guanidine-HCl extraction protocol. Aβ_40_ and Aβ_42_ levels were quantified on Meso Scale Discovery (MSD) 96-well plates by ELISA using end-specific antibodies provided by Dr. Marc Mercken (Janssen Pharmaceutica, Belgium). Monoclonal antibodies JRFcAβ40/28 and JRFcAβ42/26, which recognize the C terminus of Aβ species terminating at amino acid 40 or 42, respectively, were used as capture antibodies. JRF/AβN/25 labeled with sulfo-TAG was used as the detection antibody, and the plate was read in MSD Sector Imager 6000.

### Statistics

All data are shown as mean ± SEM. Differences between mean values were determined using 1-way or 2-way analysis of variance (ANOVA), or unpaired *t* test with Welch correction (Welch test). Time series were compared with 2-way repeated measures ANOVA (RM-ANOVA) procedures with the Holm-Sidak test for post hoc comparison. For data obtained from whole-cell patch-clamp recordings, statistical significance was determined using the Welch test or Kolmogorov-Smirnov test.

## Results

### Determination of Aβ_40_ and Aß_42_ in brains of App^NL-G-F^ and App^NL^ mice

**Fig. 1:**
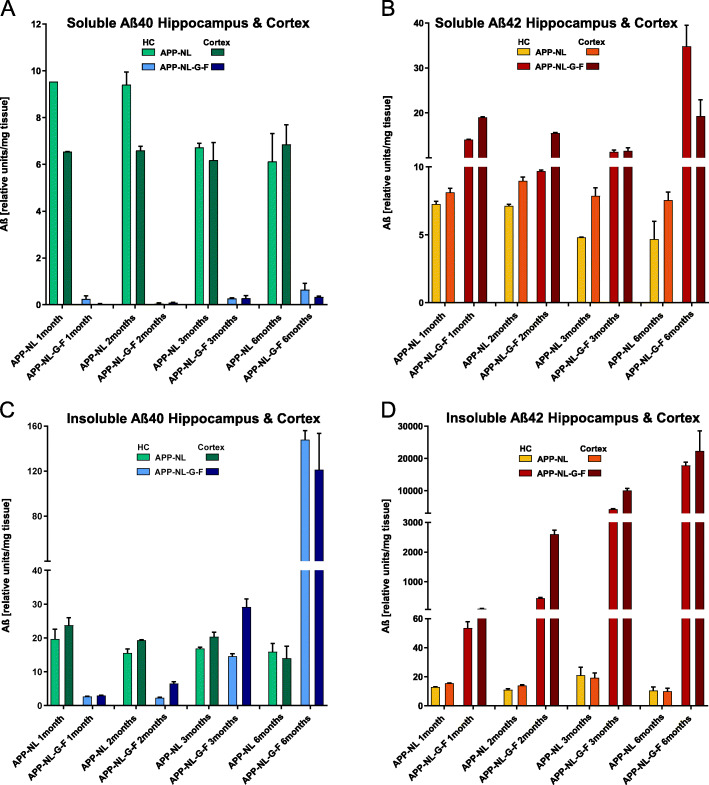
Quantification of Aβ_40_ and Aβ_42_ levels by sandwich ELISAs in the hippocampus and prefrontal cortex of *App*^*NL-G-F*^ and *App*^*NL*^ mice. **a**, **b** *App*^*NL*^ mice do not display changes of soluble Aß_40_ (**a**) and Aß_42_ (**b**) between 1 and 6 months of age. *App*^*NL-G-F*^ mice present low levels of Aß_40_ with no age-dependent changes, both in the hippocampus and cortex (**a**). While the Aβ_42_ levels in these mice are similar between 1 and 3 months, they increase in the hippocampus but not in the cortex of 6-month-old mice (**b**). **c**, **d** Insoluble Aß_40_ (**c**) and Aß_42_ (**d**) remain at similar levels in the hippocampus and cortex of *App*^*NL*^ mice across the whole age range. Interestingly, in *App*^*NL-G-F*^ mice, Aß_40_ is at plateau levels between 1 and 3 months and increases at 6 months, but levels of Aß_42_ accumulate almost linearly during aging. Of note, the increase in insoluble Aβ_42_ appears to start earlier in the cortex than in the hippocampus (**d**). Data represent mean ± SEM (*n* = 2 mice per indicated time point)

To get an indication of the age-dependent increase in the levels of soluble and insoluble Aß1-40 and Aß1-42 and to confirm previous data on the same genotypes [13], we performed ELISA measurements in a small sample of mice (*n* = 2 per genotype and age). In *App*^*NL*^ mice, no age-related changes in soluble Aβ_40_ and Aß_42_ were found in the hippocampus and cortex between 1 and 6 months (Fig. 1a, b). The levels of Aβ_40_ and Aß_42_, respectively, in both brain regions were similar. In *App*^*NL-G-F*^ animals, Aß_40_ is generated at low levels (less than a tenth compared to *App*^*NL*^ mice) without any noticeable age-dependent changes. The levels of Aß_42_ are higher than in *App*^*NL*^ mice and show similar values between 1 and 3 months but a clear upregulation in the hippocampus of 6-month-old mice. The upregulation is absent in the cortex. Insoluble Aß_40_ and Aß_42_, respectively, remain at similar levels in the hippocampus and cortex of *App*^*NL*^ mice across the whole age range, but show peptide-specific changes in the same regions in *App*^*NL-G-F*^ mice (Fig. 1c, d). Here, Aß_40_ is at plateau levels between 1 and 3 months and increases at 6 months, but levels of Aß_42_ accumulate almost linearly during aging and are more than two hundred-fold higher at 6 months than at 1 month of age. Between the ages of 2 and 3 months, the rise of Aß_42_ levels seems to be faster in the cortex than in the hippocampus. These results are consistent with previous reports [13].

### Intact synaptic function and synaptic plasticity in the hippocampus of *App*^*NL-G-F*^ mice at 3–4 months of age

**Fig. 2:**
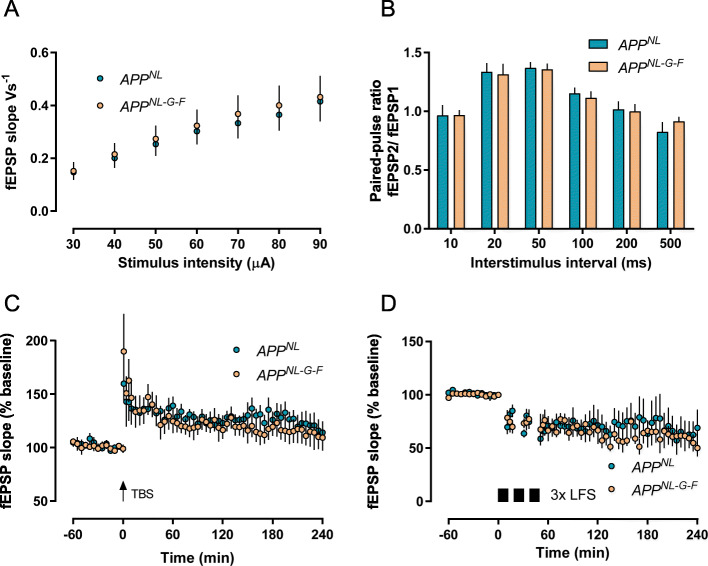
Unchanged basal synaptic transmission, short-term plasticity, and long-term plasticity in the hippocampal CA1 region of *App*^*NL*^ and *App*^*NL-G-F*^ mice at 3–4 months of age. **a** I/O curves of *App*^*NL*^ and *App*^*NL-G-F*^ mice were almost identical. **b** Paired-pulse ratio was intact at every interval tested in both groups. **c** *App*^*NL*^ and *App*^*NL-G-F*^ animals developed a robust LTP for 4 h. **d** Triple repetition of LFS stimulation induced robust LTD that did not differ between *App*^*NL*^ and *App*^*NL-G-F*^ mice. Data are shown as mean ± SEM

The hippocampus belongs to the regions that are early affected by Aß pathology in humans, subsequently to the initial infiltration of the neocortex [38]. In particular, the CA1 region of the hippocampus is very vulnerable to any synaptotoxic effects. In addition, this region is the best investigated brain area for the processes of synaptic plasticity [long-term potentiation (LTP), long-term depression (LTD)], which react very sensitively to alterations in synaptic function during AD progression [10, 11, 39] and are established models for memory formation and storage at the cellular level [40, 41]. Here, we used long-term recordings of field excitatory postsynaptic potentials (fEPSPs) in the CA1 stratum radiatum to examine whether basal synaptic transmission, short-term plasticity, LTP, and LTD, evoked in acute slices of 3–4-month-old *App*^*NL-G-F*^ mice, show any discernible differences to *App*^*NL*^ control mice. As depicted in Fig. 2a, we did neither detect any genotype differences in basal synaptic transmission (input/output curves) nor in paired-pulse responses, a measure of presynaptically mediated short-term plasticity [42] (Fig. 2b).

Next, we examined activity-dependent long-term synaptic changes. The stimulation protocol we employed to induce LTP [theta burst stimulation (TBS)] is based on the “hippocampal theta rhythm,” a large-amplitude oscillation seen in electroencephalographic recordings in the range of 4–8 Hz [30, 43, 44], and as such may be considered as better approximating the physiological conditions in vivo than high-frequency stimulation (HFS) consisting of long bursts at 100 Hz. Since we found in the past that weak tetanization protocols (single TBS) are more sensitive to detect synaptic deficits than strong protocols (e.g., 3× TBS [45];), we used a single TBS stimulation to induce LTP and obtained similar potentiation in both groups (Fig. 2c; *App*^*NL*^—10 min after TBS application 137 ± 15%, 240 min 117 ± 12%, *n* = 6; *App* ^*NL-G-F*^—10 min 150 ± 14, 240 min 127 ± 15, *n* = 6). Furthermore, triple LFS stimulation resulted in similar LTD in both groups that lasted 4 h (Fig. 2d; *App*^*NL*^—10 min post-LFS application 67 ± 6%, 240 min 49 ± 8%, *n* = 6; *App* ^*NL-G-F*^—10 min 78 ± 7%, 240 min 50 ± 8%, *n* = 5). These data clearly indicated that Aß-mediated synaptotoxic effects failed to cause any significant functional deficits in the hippocampal CA1 region of *App*^*NL-G-F*^ mice at 3–4 months of age.

### *App*^*NL-G-F*^ mice show deficits in hippocampal long-term potentiation at 6–8 months

**Fig. 3:**
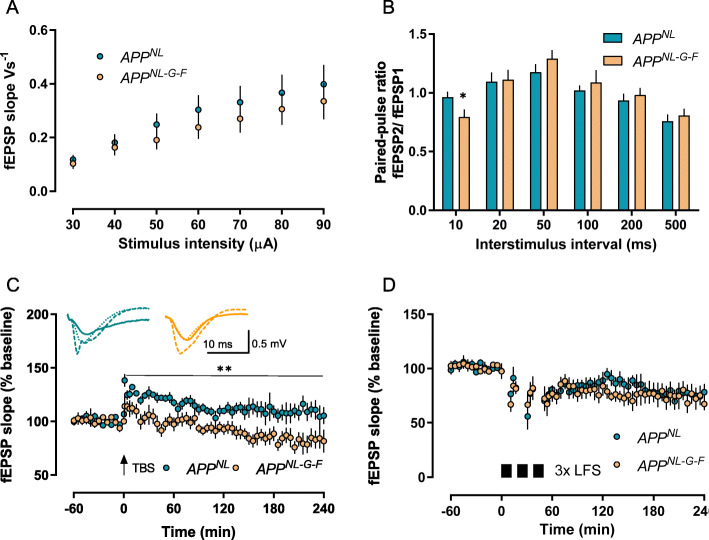
Deficits in hippocampal synaptic plasticity in *App*^*NL-G-F*^ mice at 6–8 months of age. **a** Basal synaptic transmission as measured by input/output curves did not differ significantly between genotypes. **b** Paired-pulse ratio was decreased at the 10-ms interpulse interval in *App*^*NL-G-F*^ mice compared to *App*^*NL*^ controls (*t* = 2.102, *p* = 0.0484 Welch test). **c** Slices from *App*^*NL-G-F*^ developed a smaller initial amplitude than APP NL controls and displayed impaired maintenance of potentiation resulting in a significant difference to *App*^*NL*^ littermates (*F*_1, 12_ = 12.17, *p* = 0.0045, RM-ANOVA). **d** LFS-induced LTD was not affected by Aß-mediated pathology in *App*^*NL-G-F*^ mice. Data are shown as mean ± SEM. Asterisks indicate difference in fEPSPs between *App*^*NL*^ and *App*^*NL-G-F*^: *p < 0.05, **p < 0.01. Insets depict representative analogue traces, taken during baseline recording (solid line), after TBS (broken line), and at the end of recording (240 min, dotted line)

The absence of any synaptic deficit in the CA1 region at 3–4 months of age raised the question as to whether synaptic functions are affected by increased Aβ levels at the age of 6–8 months, in particular by the marked increase in the aggregation-prone Aß_42_ detected by ELISA, which should promote the formation of synaptotoxic oligomers and aggregation. We performed, therefore, the same electrophysiological tests at 6–8 months. Although we did not find any genotype differences in basal synaptic function (Fig. 3a), there were clear deficits in *App*^*NL-G-F*^ compared to *App*^*NL*^ in other measures. First, the paired-pulse ratio was significantly reduced at 10 ms (*t* = 2.102, *p* = 0.0484 Welch test; *App*^*NL-G-F*^ *n* = 12, *App*^*NL*^ *n* = 18; Fig. 3b), suggesting short-term plasticity has been affected. More obviously, when long-term potentiation was examined, we found it significantly impaired in slices from *App*^*NL-G-F*^ (*n* = 7) animals compared to *App*^*NL*^ (*n* = 7) across 4 h of recording (Fig. 3c; main effect of *genotype* for 4 h recording post-induction: *F*_1, 12_ = 12.17, *p* = 0.0045, RM-ANOVA). The difference was already present immediately after the LTP induction during the first 10 min (*F*_1, 12_ = 6.362, *p* = 0.0268, RM-ANOVA). Thereafter, LTP of *App*^*NL*^ mice returned to baseline, but the potentiation of *App*^*NL-G-F*^ decayed even to values below.

When the LTP curves of 3–4 and 6–8 months old animals are compared by eye inspection, then it is already apparent that the magnitude of potentiation is lower in the older mice. Statistical analysis with RM-ANOVA confirmed a significant main effect of age in *App*^*NL*^ (*F*_1, 11_ = 4.990, *p* = 0.0472) and *App*^*NL-G-F*^ (*F*_1, 11_ = 10.82, *p* = 0.0072) animals.

In contrast to the deficits found in LTP at 6–8 months, LTD experiments did not reveal any significant difference between the two genotypes (*App*^*NL*^—51 min (= 1 min after third LFS train) 70.11 ± 3.97%, 240 min 78.22 ± 7.48%, *n* = 6; *App*^*NL-G-F*^—51 min 72.32 ± 11.30%, 240 min 67.31 ± 5.75%, *n* = 6; Fig. 3d).

### Impairment of synaptic plasticity starts at 3–4 months and of basal synaptic transmission at 6–8 months in PFC

**Fig. 4:**
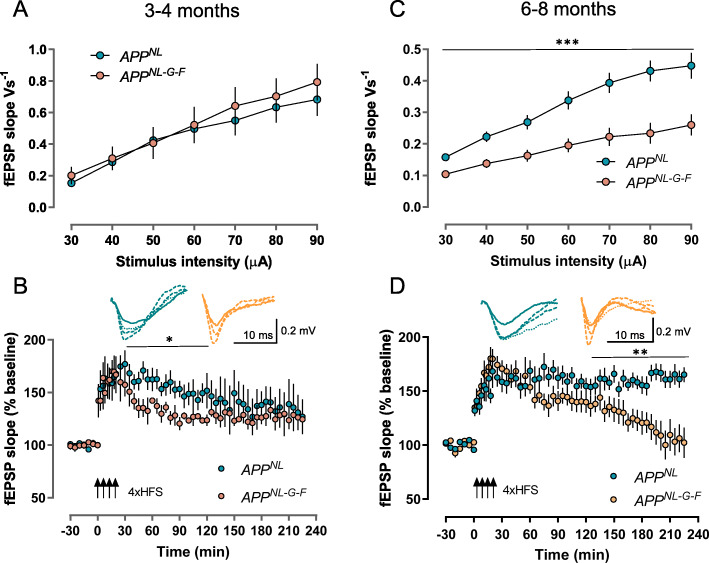
Extracellular recordings in the medial PFC of cortical slices from *App*^*NL*^ and *App*^*NL-G-F*^ mice at 3–4 (**a**, **b**) and 6–8 months of age (**c**, **d**). **a** Basal synaptic transmission is unaffected at 3–4 months as shown by the input-output curves. **b** Long-term potentiation induced by 4× HFS in the prelimbic and infralimbic region of the PFC from *App*^*NL-G-F*^ mice is of similar magnitude as from the *App*^*NL*^ littermates, but the potentiation of *App*^*NL-G-F*^ decays faster resulting in a temporary difference to *App*^*NL*^ from 30 min post-induction until 120 min (*F*_(1, 7)_ = 9.052, *p* = 0.0197, RM-ANOVA). **c** Input-output curves indicate a severe reduction in basal synaptic transmission in *App*^*NL-G-F*^ mice at 6–8 months of age (two-way RM-ANOVA *F*_(1, 15)_ = 16.82, *p* = 0.0009). **d** Although LTP of a similar initial magnitude could be induced in *App*^*NL-G-F*^ mice (*n* = 8), the potentiation was not maintained and declined to baseline. In contrast, *App*^*NL*^ mice (*n* = 8) expressed a robust LTP that remained at about the same level until the end of recordings resulting in a highly significant genotype difference (RM-ANOVA *F*_(1, 16)_ = 12.04, *p* = 0.0032). Data are shown as mean ± SEM. Asterisks indicate difference in fEPSPs between *App*^*NL*^ and *App*^*NL-G-F*^: *p < 0.05, **p < 0.01, ***p < 0.001 (RM-ANOVA). Insets depict representative analogue traces, taken during baseline recording (solid line), after HFS (long-broken line), 90 min after LTP induction (broken line), and at the end of recording (225 min, dotted line). Note that the analogue traces of *App*^*NL-G-F*^ mice at 90 min and 225 min in **b** are exactly on top of each other

In the second set of experiments, medial PFC field potentials were evoked in cortical brain slices from 3–4- (Fig. 4a, b) and 6–8-month-old (Fig. 4c, d) *App*^*NL*^ and *App*^*NL-G-F*^ mice. The prefrontal cortex (PFC) is one of the areas that are very susceptible to amyloid pathology and first affected by it [38, 46]. Reduced synaptic density in PFC is considered as one of the strongest pathological correlates of cognitive decline and the severity of dementia [46–48]. A recent study reported significant microstructural changes in the PFC of 9–10-month-old *App*^*NL-G-F*^ mice, which were examined by diffusion tensor magnetic resonance imaging [49].

At the age of 3–4 months, we did not find significant differences in the input-output curves (Fig. 4a). However, the LTP recordings of *App*^*NL-G-F*^ mice (*n* = 4) decayed faster after induction as compared with *App*^*NL*^ (*n* = 5) animals resulting in a temporary significant difference from 30 min post-induction until 120 min (*F* (1, 7) = 9.052, *p* = 0.0197, RM-ANOVA; Fig. 4b). Thereafter, the LTP values of both groups converged to similar values.

At 6–8 months, *App*^*NL-G-F*^ (*n* = 8) mice were even more severely impaired, because they showed a pronounced reduction in basic synaptic transmission compared to *App*^*NL*^ (RM-ANOVA *F*_(1, 15)_ = 16.82, *p* = 0.0009; Fig. 4c) and LTP decayed to baseline (Fig. 4d). Since LTP of *App*^*NL*^ (*n* = 10) was robustly maintained, this led to a significant genotype difference from 120 min after LTP induction until the end of recording (RM-ANOVA *F*_(1, 16)_ = 12.16, *p* = 0.0041).

In addition to the “in-between” genotype differences in basal synaptic transmission and LTP at the two ages investigated, we tested also whether there was also an age-dependent “within” genotype change in basal synaptic transmission. Statistical comparison with RM-ANOVA confirmed a significant age difference for *App*^*NL-G-F*^ mice (*F*_1, 12_ = 14.26, *p* = 0.0026) while not reaching statistical significance in *App*^*NL*^ littermates (*F*_1, 13_ = 4.196, *p* = 0.0613).

### Increased frequency of excitatory and inhibitory miniature postsynaptic currents in *App*^*NL-G-F*^ mice

The deficits in synaptic plasticity in *App*^*NL-G-F*^ mice indicated alterations in the complex machinery of the trans-synaptic signaling regulating crucial mechanisms like neurotransmitter release and recycling, postsynaptic receptor binding, and the balance between glutamatergic and GABAergic activity. In order to investigate such changes in more detail, i.e., at the level of elementary synaptic function, we performed patch-clamp whole-cell recordings of miniature excitatory postsynaptic currents (mEPSCs) and miniature inhibitory postsynaptic currents (mIPSCs), consecutively from the same CA1 pyramidal neurons [35–37] of 6–8-month-old mice.

**Fig. 5:**
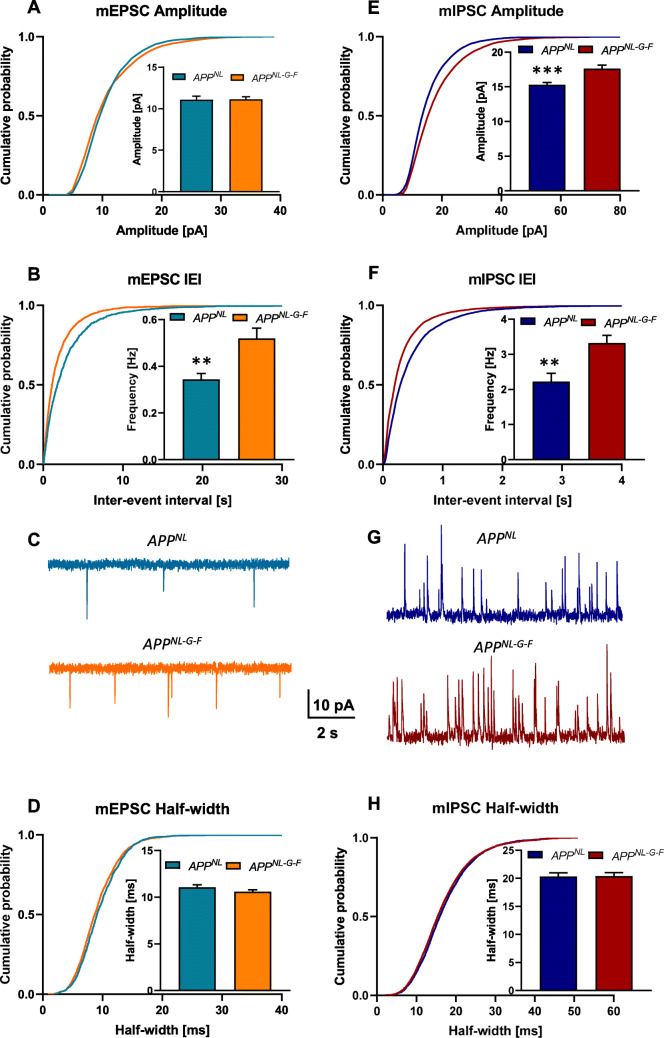
Analysis of action potential-independent mEPSCs (**a**–**c**) and mIPSCs (**d**–**f**) in 6–8 months old *App*^*NL*^ and *App*^*NL-G-F*^ mice. **a**, **b** The mean frequency but not the amplitude of mEPSCs is markedly increased in *App*^*NL-G-F*^ mice as compared to *App*^*NL*^ control animals (see insets in **a** and **b**, *p* = 0.0012 Welch test). **c** Representative traces from mEPSC recordings illustrate the higher mEPSC frequency of CA1 pyramidal neurons from *App*^*NL-G-F*^ mice compared to *App*^*NL*^ controls. **d** mEPSC half-width, a measure that characterizes the kinetics of inactivation, is unchanged in *App*^*NL-G-F*^ mice. Analyses of the probability distributions of the data (**a**, **b**, **d**) revealed significant genotype differences for all three parameters. **e**, **f** Both mean amplitude and mean frequency of mIPSCs show a pronounced increase in *App*^*NL-G-F*^ mice [amplitude *App*^*NL-G-F*^ (inset in **e**, *p* = 0.0003 Welch test), frequency (inset in **f**, *p* = 0.0013 Welch test)]. **g** Representative traces from mIPSC recordings illustrate the marked increase in the mIPSC frequency of CA1 pyramidal neurons from *App*^*NL-G-F*^ mice compared to *App*^*NL*^ controls. **h** The kinetics of inactivation as measured by the half-width of mIPSCs was the same in both genotypes. Kolmogorov-Smirnov tests yielded significant genotype differences for the amplitude, the frequency, and the half-width of mIPSCs. Asterisks indicate difference between *App*^*NL*^ and *App*^*NL-G-F*^: **p < 0.01, ***p < 0.001

Our measurements revealed a marked increase in the mean frequency of mEPSCs in *App*^*NL-G-F*^ mice as compared to *App*^*NL*^ control animals (inset in Fig. 5b, *App*^*NL-G-F*^—0.519 ± 0.044 Hz, *n* = 8; *App*^*NL*^—0.344 ± 0.025 Hz, *n* = 6, *p* = 0.0012 Welch test). In contrast, we did not find any significant change in the mean amplitude or half-width of mEPSCs (insets in Fig. 5a, c).

Thereafter, we examined the same parameters for mIPSCs and found here not only a pronounced increase in the mean frequency of mIPSCs in *App*^*NL-G-F*^ (inset in Fig. 5e, *App*^*NL-G-F*^—3.321 ± 0.371 Hz, *n* = 8; *App*^*NL*^—2.225 ± 0.311 Hz, *n* = 6, *p* = 0.0430 Welch test) but also significantly higher values of the mean IPSC amplitude in *App*^*NL-G-F*^ (inset in Fig. 5d, *App*^*NL-G-F*^—17.65 ± 0.9069 pA, *n* = 8; *App*^*NL*^—15.31 ± 0.3647 pA, *n* = 6, *p* = 0.0403 Welch test). As with the mEPSCs, there was no difference between genotypes in mIPSC half-width.

In-depth analyses of the probability distributions of the data (Fig. 5a–f) with the Kolmogorov-Smirnov test confirmed genotype differences for those parameters that had significantly different mean values as mentioned above. However, it detected also significant genotype differences in the cumulative probability distribution of several other parameters (mEPSC amplitude, mEPSC inter-event interval, mIPSC amplitude, mIPSC inter-event interval, mIPSC half-width: all *p* < 0.0001; mEPSC half-width *p* = 0.0033.

## Discussion

The study of genetically modified animals, mainly transgenic and knock-out mouse models, has been invaluable in the past to gain significant insights into the pathological mechanisms of AD. Although none of these models fully reproduces the complete spectrum of AD, they recapitulate major aspects of the disease [10] and their detailed characterization at the molecular, cellular, network, and behavioral levels is important to understand the utility and limitations of each model [50]. The *App*^*NL-G-F*^ mouse model, investigated in our study, belongs to the “second-generation” mouse models produced with the hope that they would display greater concordance between preclinical animal studies and human clinical trials. Aß-mediated pathology (as becoming overt by plaque deposition) starts already at the age of 2 months, and the cortex is almost saturated at 7 months. Likewise, neuroinflammation and changes in synaptic proteins, which are two of the main AD hallmarks, are also observed in these mice at early stages [13]. *App*^*NL*^ mice were used as control in our experiments, because they are generated by the same knock-in strategy as *App*^*NL-G-F*^ mice and show the same increase in the level of the C-terminal fragment β (CTF-β) as the latter, due to the Swedish mutation.

Here, we used an electrophysiological approach to gain insights into putative functional deficits at different levels of synaptic function, and examined basal synaptic transmission, synaptic plasticity, and miniature synaptic currents. Although there is an increasing awareness of AD as a multidimensional and multicellular process [9, 12], many of the pathological processes involved in AD that operate in parallel and/or interactively in neurons and other cells in the brain inevitably converge on the cellular mechanism underlying memory and compromise them in a complex manner [1, 20]. Thus, synapses and synaptic plasticity are major downstream sites and mechanisms of pathophysiological convergence [21, 22]. We recorded from the hippocampus and prefrontal cortex since these two brain regions are closely linked anatomically and their bidirectional functional interaction is essential for higher-order cognitive functions, including the encoding and retrieval of working, emotional, and episodic types of memory [51–53]. The synchronization of hippocampal and prefrontal neural activity allows the coordinated hippocampal-prefrontal replay which is considered a key mechanism for memory consolidation [54]. Importantly, the hippocampus (in particular, its CA1 region) and the PFC are highly susceptible to amyloid pathology, in particular to the synaptotoxic effects of soluble low-n Aß oligomers ([55–57]; see [3] for critical discussion).

In the present study, synaptic functioning was inspected at 3–4 and 6–8 months of age. Evidence for a beginning impairment was detected for PFC LTP in 3–4 months old *App*^*NL-G-F*^ mice, which was further aggravated at 6–8 months of age. The only other study of PFC LTP in an AD mouse model by Battaglia et al. reported an LTP deficit in the PFC of transgenic APP/PS1 mice, but did not specify the age [58]. In the hippocampal CA1 region, a deficit in LTP was only recognizable in 6–8 months old *App*^*NL-G-F*^ animals. The deficits in HC and PFC LTP were most apparent in the maintenance of potentiation and less in the initial magnitude, because LTP in both regions decayed to baseline values or even below, as observed in HC. Given the length of our LTP recordings of more than 3 h, we found late-LTP being impaired (≥ 3 h after induction), whose mechanisms have been implicated in long-term memory [59, 60]. Late-LTP was demonstrated to be highly susceptible to disruption by AD pathology but was rarely recorded in AD animal models in vitro in the past [61–64]. The earlier impairment of LTP in PFC compared to HC in *App*^*NL-G-F*^ mice is likely caused by a higher Aß_42_ burden and/or its earlier onset in this region at an early age, as indicated by the results of the ELISA measurements and further supported by significantly higher immunoreactive plaque areas in the PFC vs. HC of *App*^*NL-G-F*^ mice at the two ages investigated here [13]. According to Benilova et al. and Yang et al., amyloid plaques likely sequester soluble Aß oligomers and release them under certain conditions [3, 57]. Thus, a dynamic equilibrium between toxic oligomers and inert fibrils might exist around the plaques, resulting in the local release of neurotoxic oligomers into the surrounding tissue. According to this scenario, the higher plaque load in the cortex compared to the hippocampus of *App*^*NL-G-F*^ mice is likely to result in a higher tissue concentration of toxic oligomers in the cortex which would explain the earlier onset of synaptic deficits, as detected by a faster decay of LTP at the age of 3–4 months. Interestingly, in HC but not PFC, we found a significant reduction in the magnitude of potentiation with age. This difference was bigger in *App*^*NL-G-F*^ mice suggesting a major contribution of Aß-driven pathology to the age-dependent decline in potentiation.

In contrast to the pronounced impairment of LTP, NMDAR-dependent LTD induced by LFS at 2 Hz [32, 65] was not affected in *App*^*NL-G-F*^ mice. While LTP has been extensively studied in transgenic and knock-out animal models of AD [10, 11, 27], LTD as the physiological counterpart of LTP was largely neglected. Most of these AD-related LTD studies focused on the effects of amyloid ß-protein and its fragments. While some studies reported the resistance of LTD to the application of Aß peptides or to the pathology-driven increase of their endogenous levels [66], most studies found an increase in LTD or a facilitation of its induction [26, 56, 67–71]. The reasons for the missing deficits in LTD are not clear. It is unlikely that 2 Hz LTD is completely insensitive to Aß-mediated pathology because we obtained a clear impairment in 12-month-old APP/PS1 mice (unpublished laboratory data). Impaired LTD was also described for the cerebellum of APP/PS1 mice [72]. Thus, LTD is sensitive to Aß-mediated pathology, but compared to LTP, LTD may require higher levels of soluble Aß oligomers to get impaired [66, 73].

It is well accepted that (several forms of) LTP and LTD require the activation of NMDA receptors and the increase of intracellular calcium, but LTP and LTD are predominantly expressed and sustained by different signaling pathways. The lack of effects of Aß-mediated pathology on LTD in *App*^*NL-G-F*^ mice therefore indicates that Aß oligomers exert differential effects on signaling cascades that sustain LTP and LTD, respectively. This supposition is supported by published evidence. For example, Aβ oligomers block the accumulation of CaMKIIα, a central kinase in bidirectional synaptic plasticity, at excitatory synapses during LTP induction but not at inhibitory synapses during LTD [74]. Furthermore, Kootar et al. revealed by ex vivo experiments in hippocampal slices that the LTP impairment by Aß oligomers requires the functional crosstalk with glucocorticoid receptors, but the Aß oligomer-mediated LTD induction does not [75].

In addition to the marked deficit in LTP in HC and PFC at 6–8 months of age, basal synaptic transmission in PFC was significantly impaired in *App*^*NL-G-F*^ mice, as indicated by the lower input/output curve. While Battaglia et al. did not find a difference in basal synaptic transmission in the PFC of the transgenic APP/PS1 model [58], Roder et al. reported reduced basal synaptic transmission in the PFC of 6-month-old APP23 mice [76]. Such a severe disturbance of synaptic functioning was reported also for some transgenic AD mouse models like APP23, APPLd2, APPind H6, hAPPJ9, hAPPJ20, PS1M146V KI, and 3xTg ([76], see [27] for detailed references) in the hippocampal CA1 region, but in *App*^*NL-G-F*^ mice, we did not detect any significant reduction of basal synaptic transmission in this region.

The absence of any overt deficits in basal synaptic transmission and synaptic plasticity in *App*^*NL-G-F*^ at 3–4 months might be due to a composition and/or levels of Aß_42_ and Aß_40_ oligomers at this age that were not yet sufficient to exert discernible synaptotoxic effects. One of the reasons could be that the plaques that already exist at this age [13] sequester soluble high-n oligomers, a putative source for toxic low-n oligomers [57], to a higher degree at early than later stages of pathology.

Further of note, when we checked short-term plasticity by paired-pulse stimulation [77], we found a significantly lower value for *App*^*NL-G-F*^ mice at an interpulse interval of 10 ms. In previous studies of transgenic AD mouse models, significant changes in the paired-pulse ratio (PPR) were very rarely detected [27]. Since the interval of 10 ms is an integral part of the 100-Hz bursts in the TBS protocol that was used in our study to induce LTP, the decay of responses to repeated stimuli resulted presumably in a lower efficacy of the TBS protocol, leading to the observed LTP deficit in *App*^*NL-G-F*^ animals. A similar relationship between decreased paired-pulse response at 10 ms and defective LTP was previously described in neurogranin knock-out mice, provided a weak stimulation protocol was employed [78, 79]. The found reduction of the paired-pulse ratio at 10 ms is most likely due to increased GABA_A_ergic inhibition, because it could be rescued in previous experiments by application of GABA_A_ receptor antagonists such as picrotoxin [33].

In order to better understand at an elementary level to which extent pre- vs. postsynaptic changes and a shift in the balance between glutamatergic excitatory and GABAergic inhibitory activity contribute to the disturbances in synaptic function, we measured action potential-independent miniature EPSCs (mEPSCs) and miniature IPSCs (mIPSCs). The obtained significant changes in the mean frequency of mEPSCs in *App*^*NL-G-F*^ mice, together with an unchanged mean amplitude and half-width, suggest an increased presynaptic activity of glutamatergic synapses in response to the progressing Aß-mediated pathology. In contrast to our finding, Chang et al. observed in 2xKI mice that carry the Swedish mutation and the P264L presenilin 1 mutation but do not overexpress APP an age-dependent reduction in mEPSC amplitude [80]. Likewise, D’Amelio et al. found in 3-month-old Tg2576 transgenic mice a downregulation of mEPSC frequency [26].

The overall effect of synaptic pathology on mIPSCs in *App*^*NL-G-F*^ mice seems to be even stronger because we did not only find an increased mean frequency of mIPSCs in *App*^*NL-G-F*^ but also a significantly higher mean IPSC amplitude, i.e., synergistically acting pre- and postsynaptic mechanisms. The apparent increase in elementary inhibitory activity could be involved in the deficits in LTP induction at this age and is reminiscent of reported compensatory inhibitory mechanisms that developed in 4–7 months old hAPP-J20 mice in response to neuronal overexcitation [25]. These mice, carrying the Swedish and Indiana FAD mutations, show an enhanced frequency of mIPSCs similar to our findings in *App*^*NL-G-F*^ mice. In addition, the amplitude of large-amplitude mIPSCs was found to be increased in this study.

## Conclusions

Taken together, the characterization of elementary synaptic functions and long-term synaptic plasticity in *App*^*NL-G-F*^ mice seems to point to an apparent synaptic enigma in these mice. The marked impairment of primarily postsynaptic processes at the level of synaptic plasticity contrasts with the found upregulation of presynaptic processes in elementary (miniature) synaptic function. Major changes in presynaptic function and markers are not unique to APP-KI mice; they were also found in AD patients [81–83]. Thus, the found upregulation is most likely the result of a compensatory “homeostatic” response. Such a mechanism could serve to counteract impairments of synaptic plasticity caused by postsynaptic deficits in order to confine the overall magnitude of AD-driven imbalances in synaptic function. The described changes in miniature pre- and postsynaptic mechanisms in concert with defects in synaptic plasticity are expected to stimulate further research, paving the way for new therapeutic strategies that target the vulnerable synaptic machinery that is central to AD-mediated cognitive decline.

## Limitations

The current study was designed to perform a first characterization of synaptic plasticity and other synaptic functional readouts of two new second-generation AD mouse models (*App*^*NL-G-F*^ and *App*^*NL*^ mice), which were produced by a knock-in strategy with the hope that they would display greater concordance between preclinical animal studies and human clinical trials. Indeed, recent studies support the supposition that they can serve as valuable models to examine Aß-related pathology in “preclinical AD.” The *App*^*NL-G-F*^ mice harbor a humanized APP construct that contains three mutations associated with familial Alzheimer’s disease: the Swedish, the Beyreuther/Iberian, and the Arctic mutation in the APP gene. These three mutations increase total Aß production, enhance the Aβ_42_/Aβ_40_ ratio, and promote Aß aggregation, respectively. *App*^*NL*^ mice carrying only the Swedish mutation serve as control. Of note, in *App*^*NL-G-F*^ mice, Aβ deposition starts already at 2 months and is nearly saturated by 7 months.

Against this background, we decided to investigate the two genotypes at two different stages of pathology, at an age of 3–4 months with mild to moderate Aß expression and an expected absence of major functional phenotypes, and at an age of 6–8 months with aggravated pathology, for which we anticipated discernible functional deficits in synaptic plasticity and other functional synaptic readouts. At the latter age, *App*^*NL-G-F*^ are still devoid of marked behavioral phenotypes according to published studies. Because our synaptic measurements are done in vitro, we have to use different batches of mice for this work. Given the attractiveness of these new mouse AD models, there was a high demand by several groups at KU Leuven to use them for a variety of methodological studies. This sometimes led to a bottleneck in breeding in the animal facility, making it difficult to receive batches of mice with the same sample size for all experiments.

For the statistical design of the study, we focused on pathophysiologically meaningful deficits, i.e., a rather large effect size and used homozygous females. Because we are conducting electrophysiological recordings of long-term potentiation (LTP) and long-term depression (LTD) with a sampling interval of 5 min, we are dealing with time series and use, therefore, repeated measures ANOVA (RM-ANOVA) as the standard of our analysis, with group/treatment as between-subject factor and time as within-subject factor. Due to the sensitivity of synaptic plasticity measures (e.g., LTP) towards pathological deteriorations, we observe usually rather big effect sizes. Therefore, the study has been powered to detect between-group differences, assuming an effect size *F* = 0.95, using an alpha err probe = 0.05, power (1-ß err probe) = 0.80, and a correlation between repeated measures of 0.85. Our common recording time of LTP or LTD is 3 to 4 h with a sampling interval of 5 min, resulting in a high number of repetitions in RM-ANOVA. In the event that we look at particular phases (periods) of LTP or LTD (e.g., early LTP, late-LTP), we work at least with periods of 90 min, which is equal to 19 repetitions in RM-ANOVA. Using these values in G-Power, we came to a minimal required total sample size of the two groups of 10 (5 per group), which was fulfilled throughout our measurements, except the 3-month-old *App*^*NL-G-F*^ group in the PFC recordings, where we lost the data of one animal due to technical problems during recording.

In terms of the reproducibility of the data, repeating the experiments was not an option as the staff who performed most of the measurements (a doctoral student, a postdoc) had to leave the laboratory because their contracts were running out and could not be renewed and new staff could not be hired because of the difficult local grant situation. However, according to our experience, it seems to be more important that new findings are replicated independently by other laboratories under (usually) slightly different methodical conditions to get a more robust picture of the particular phenotype. Thus, we noticed often in the past that results could be precisely replicated under the same conditions in one laboratory while other laboratories failed consistently to confirm them. Prominent examples for the latter are the controversies about the “molecular switch” in the field of mGluR receptors, the role of NR2A and NR2B NMDA receptor subunits in synaptic plasticity and learning, and the function of PKMzeta within the same functional circuits. Here, the laboratories that first described the function could consistently reproduce the initial finding, while other laboratories continued to fail. In relation to the present study, it would therefore be best if the same or similar experiments were carried out in other laboratories in order to check/validate the reproducibility of the presented results under different laboratory conditions.

## Data Availability

The datasets used and/or analyzed during the current study are available from the corresponding author on reasonable request.

## Abbreviations

Aß: Amyloid ß
ACSF: Artificial cerebrospinal fluid
AD: Alzheimer’s disease
ANOVA: Analysis of variance
APP: Amyloid precursor protein
*App*^*NL-F*^: Knock-in mouse model of sporadic AD co-expressing the Swedish (KM670/671NL) mutation and the Beyreuther/Iberian (I716F) mutation
*App*^*NL-G-F*^: Knock-in mouse model of sporadic AD carrying the Swedish mutation, the Beyreuther/Iberian mutation, and the Arctic (E693G) mutation
KI: Knock-in
CNQX: 6-Cyano-7-nitroquinoxaline-2,3-dione
CTF-β: C-terminal fragment β
D-APV: d-Aminophosphonovalerate
FAD: Familial forms of AD
fEPSPs: Field excitatory postsynaptic potentials
HFS: High-frequency stimulation
LFS: Low-frequency stimulation
LTD: Long-term depression
LTP: Long-term potentiation
LOAD: Late-onset AD
mEPSCs: Miniature excitatory postsynaptic currents
mIPSCs: Miniature inhibitory postsynaptic currents
MSD: Meso Scale Discovery
NFTs: Neurofibrillary tangles
PBS: Phosphate-buffered saline
PFC: Prefrontal cortex
PPR: Paired-pulse ratio
*PSEN1*: Presenilin 1
*PSEN2*: Presenilin 2
RM-ANOVA: Two-way repeated measures ANOVA
TBS: Theta burst stimulation
Welch test: Unpaired *t* test with Welch correction
